# Affiliate stigma and health-related quality of life among caregivers of people with severe mental illness in a collectivist context: a cross-sectional study

**DOI:** 10.3389/fpsyt.2026.1805823

**Published:** 2026-06-12

**Authors:** Peng Fu, Ziyi Xiong, Yang Liu, Yong Li, Lian Yang

**Affiliations:** 1 School of Public Health, Chengdu University of Traditional Chinese Medicine, Chengdu, Sichuan, China; 2 Healthcare Evaluation and Organizational Analysis (HEOA) Group, School of Public Health, Chengdu University of Traditional Chinese Medicine, Chengdu, Sichuan, China

**Keywords:** affiliate stigma, health-related quality of life, influencing factors, primary caregivers, severe mental disorders

## Abstract

**Background:**

Caregivers of individuals with severe mental disorders often face a dual burden of affiliate stigma and reduced health-related quality of life (HRQoL). However, local evidence regarding the current status of these challenges and their interrelationship remains limited.

**Methods:**

This cross - sectional study examined the level of affiliate stigma and its association with HRQoL among primary caregivers of individuals with severe mental disorders. In 2024, a total of 297 primary caregivers from Sichuan Province, China, were surveyed using the Chinese version of the Affiliate Stigma Scale and the EuroQol Five-Dimensional Questionnaire - Three-Level version (EQ-5D-3L) questionnaire. Additionally, sociodemographic data were collected. Univariate analysis and Tobit regression were utilized for statistical analysis.

**Results:**

The mean affiliate stigma score among caregivers was 48.67 ± 15.63. The mean EQ-5D utility index was 0.95 ± 0.07, and the mean Visual Analog Scale self-rated health score was 70.48 ± 17.22. Univariate analyses revealed that HRQoL was significantly associated with age, income, gender, educational level, and occupational status. Multivariable Tobit regression analysis indicated that affiliate stigma was a significant negative predictor of caregiver HRQoL (EQ-5D-3L: *β* = - 0.0032, p < 0.001; EQ-VAS: *β* = - 0.3175, p < 0.001). Additionally, female caregivers exhibited significantly lower utility index scores, and lower income was significantly associated with lower self-rated health scores (the former EQ-5D-3L: *β* = - 0.0295, p < 0.05; the latter EQ-VAS: *β* = 7.6221, p < 0.001).

**Conclusion:**

This study demonstrated a significant negative association between affiliate stigma and HRQoL among primary caregivers of individuals with severe mental disorders. These findings highlight the need for targeted interventions to reduce affiliate stigma and improve the health - related quality of life and overall physical and psychological well-being of caregivers.

## 1 Introduction

Severe mental disorders impose substantial challenges on affected patients and their entire families. Family members often serve as the primary providers of long-term care. Family caregivers frequently bear extensive caregiving responsibilities while enduring significant yet usually overlooked psychological burdens. One such burden is affiliate stigma, defined as the psychological process by which caregivers closely associated with a stigmatized person internalize societal negative attitudes toward that person, leading to feelings of shame, self-blame, and self-devaluation (1). In China, affiliate stigma among caregivers is particularly prevalent, with studies reporting that up to 78.99% of caregivers experience significant affiliate stigma (2). A study targeting primary caregivers of adult patients with severe mental disorders in Addis Ababa, reported that 54.9% of caregivers experienced affiliate stigma (3). Notably, this internalized stigma is not a static burden but a key factor in a vicious cycle.

Globally, the disease burden of mental disorders remains severe, and family members constitute the primary source of care for patients with severe mental disorders. According to the World Health Organization, over one billion people worldwide are affected by mental disorders, including approximately 60 million individuals living with schizophrenia or bipolar disorder (4). In China, the lifetime prevalence of mental disorders among adults is 16.6%, implying a vast population of family caregivers (5). Each affected individual represents an entire family unit, accompanied by at least one primary caregiver who bears a heavy caregiving burden. Health-related quality of life (HRQoL) is a multidimensional concept encompassing physical, psychological, and social functioning. It is widely regarded as a core indicator of overall health status (6). Previous studies demonstrated that affiliate stigma may increase caregivers’ vulnerability to depression, thereby reducing their HRQoL, compromising caregiving capacity, increasing the risk of patient relapse, and further exacerbating affiliate stigma, thus perpetuating a vicious cycle (7–9).

From a psychological perspective, internalized stigma, characterized by shame, self - blame, and feelings of worthlessness, can directly contribute to emotional distress, including depression and anxiety, which are core components of HRQoL (10). From a behavioral perspective, caregivers experiencing affiliate stigma may adopt avoidance and concealment strategies, withdrawing from social interactions and underutilizing available support services, thereby reducing social support (11, 12). Additionally, the chronic stress associated with internalized stigma may activate physiological stress responses, increasing vulnerability to somatic symptoms and physical health problems (13). In a culture centered on family collectivism, as in China, individuals do not exist independently but are deeply embedded within family, clan, and kinship networks. Family collectivism is defined as a value orientation in which individuals prioritize family interests (14). An individual’s behavior and reputation no longer belong solely to themselves but are regarded as the collective face of the entire family. When a family member is socially labeled as negative, abnormal, or problematic due to mental illness, external discrimination and prejudice extend to the entire family. This cultural backdrop informs our interpretation of the findings and helps situate the study within the broader literature on cross-cultural stigma, without treating collectivism as an empirically measured variable. Therefore, examining the status and determinants of affiliate stigma and HRQoL among primary caregivers is essential for breaking this cycle, alleviating the caregiving burden, and improving patient prognosis.

Although studies conducted in developed countries have consistently shown that higher perceived stigma among caregivers is often associated with poorer quality of life (15), several limitations in the literature remain. First, most existing quantitative studies have been conducted in Western individualistic cultural contexts, limiting the applicability of findings to collectivist societies such as China, where familial interdependence and culturally specific manifestations of stigma may fundamentally shape caregivers’ experiences (16). Second, few studies have quantitatively examined the association between affiliate stigma and HRQoL using validated instruments specifically among primary caregivers of individuals with severe mental disorders in a Chinese collectivist context. This gap is particularly significant given that over 80% of individuals with mental disorders reside in low- and middle-income countries, where family-based caregiving is the norm (17). Methodologically, some studies rely on qualitative methods or low-sensitivity instruments, leading to imprecise quantification of affiliate stigma and HRQoL (18). To address these gaps, this study conducted a cross-sectional survey of primary caregivers for individuals with severe mental disorders in Sichuan Province, jointly employing the Affiliate Stigma Scale, specifically developed to assess affiliate stigma, and the EuroQol Five-Dimensional Questionnaire - Three-Level version (EQ-5D-3L) (supplemented by the overall health visual analog scale, the EuroQol Visual Analog Scale (EQ-VAS)).

Relevant domestic research in China remains at an early stage and often focuses on caregivers of patients with specific diseases, thereby limiting the generalizability of findings (19, 20). A study by Zhang et al. in Yunnan Province demonstrated that internalized stigma among caregivers of individuals with schizophrenia is positively associated with depression and increased risk of reduced quality of life (8). Additionally, a previous study by Deng et al. on caregivers in rural China for individuals with schizophrenia demonstrated that affiliate stigma is negatively correlated with the psychological and social dimensions of caregiver quality of life (21). To provide a more comprehensive understanding of the overall caregiver experience, this study adopted a broader perspective by examining caregivers of individuals with severe mental disorders as a whole, rather than focusing on a specific disease.

Sichuan Province, located in southwestern China, has a population exceeding 83 million. Additionally, with its large population base, it is one of the regions in China with a relatively high number of individuals with severe mental disorders, with approximately 437,400 registered cases (22, 23). The combination of heavy disease burden and relatively limited mental health resources places substantial and sustained pressure on family caregivers in this region (24–26). Moreover, within the Chinese socio-cultural context, where familial collectivism and the concept of “face” are highly emphasized, mental illness is often perceived as reflecting negatively on the entire family, exposing caregivers to more complex internalized shame and social pressure (27). Therefore, conducting this survey in Sichuan Province is highly representative. This study aims to investigate the levels of affiliate stigma and HRQoL among primary caregivers of individuals with severe mental disorders in Sichuan Province, analyze their association, and provide a theoretical basis for developing effective interventions for this group.

## 2 Methods

### 2.1 Data sources

A cross-sectional survey was conducted between August and September 2024 in Sichuan Province, China. A multistage stratified cluster random sampling method was employed. Based on regional economic development (urban versus rural areas), geographic location (eastern, central, and western Sichuan), healthcare resource availability, and population distribution, six districts/counties (prefecture-level cities) in Sichuan Province were selected as sample areas: Wenjiang District and Chenghua District in Chengdu (representing urban Chengdu, the provincial capital with higher economic development and better healthcare resources), Santai County in Mianyang (representing rural areas in northern Sichuan), Guang’an District in Guang’an (representing eastern Sichuan), Fushun County in Zigong (representing southern Sichuan), and Xuzhou District in Yibin (representing western Sichuan). This geographic coverage spans more than half of the province’s administrative regions and includes both urban and rural populations, thereby improving the representativeness of the caregiver sample. The sampling frame consisted of all registered caregivers of individuals with severe mental disorders within the catchment areas of the selected community health centers and township health centers. In the first stage, one community health center and one township health center affiliated with psychiatric medical institutions were selected in each district/county (As Chenghua District has no township health center, two community health centers were selected in this district; correspondingly, two township health centers were selected in Wenjiang District, Chengdu). In the second stage, within the jurisdiction of the above primary healthcare institutions, 50 caregivers of patients with severe mental disorders were selected from each area as study participants. A scientifically designed questionnaire was administered. All investigators received standardized training before data collection and conducted face-to-face interviews with eligible caregivers of patients with severe mental disorders in the sample areas.

### 2.2 Sample and data preprocessing

The inclusion criteria were as follows: (1) patients who met the diagnostic criteria for severe mental disorders according to either the CCMD-3, or the ICD-10.Severe mental disorders generally refer to a group of mental illnesses that severely and chronically impair individuals’ daily functioning, including six major categories: schizophrenia, paranoid psychosis, schizoaffective disorder, bipolar disorder, mental disorders due to epilepsy, and mental disorders associated with intellectual disability; (2) patients who had resided in the survey area for at least 6 months; (3) All family members of the patients were identified as the patient’s primary caregiver: defined as family members who had provided care for or co-resided with the patient for at least one year prior to hospitalization, and who were primarily responsible for the patient’s daily care, including assistance with activities of daily living. If only one caregiver was identified that individual was considered the primary caregiver. If multiple caregivers were involved, the individual who spent the most time providing care was designated as the primary caregiver; (4) family caregivers who can complete questionnaires and interviews, provide informed consent, and voluntarily participate in the study. The exclusion criteria were as follows: (1) participants who had resided in the survey area for less than 6 months; (2) participants who refused or could not cooperate with study procedures. To minimize estimation bias, questionnaires with missing values were excluded during quality control procedures.

After data entry, the completeness and authenticity of the data were verified, and logical error corrections were promptly performed. Subsequently, coding errors were corrected. Invalid data were excluded based on the following criteria: (1) cases where the primary diagnosis was not severe mental disorder; (2) cases with missing or incomplete records for key variables; (3) cases with obvious logical errors in data relationships that could not be corrected through logical adjustment; (4) cases containing abnormal values. After data screening, 297 valid questionnaires were included in the final analysis.

### 2.3 Study measures

#### 2.3.1 Affiliate stigma

The Affiliate Stigma Scale (ASS), initially developed by Mak and Cheung (1), was utilized to measure stigma experienced by family caregivers of individuals with mental illness or intellectual disability (1). The ASS consists of 22 items across three dimensions: cognitive, affective, and behavioral. Responses are rated on a 4-point Likert scale from “strongly disagree” to “strongly agree,” yielding a total score of 22 - 88. Higher scores indicated greater levels of affiliate stigma among caregivers. The ASS has demonstrated excellent reliability in previous studies. Wong et al. reported a Cronbach’s alpha of 0.96 among family caregivers of individuals with schizophrenia (28). The Chinese version, translated and culturally adapted by Chang et al., showed strong psychometric properties with a Cronbach’s alpha of 0.929 (29). More recently, Yin et al. further refined the scale to better align with Chinese linguistic conventions and applied it to measure stigma among parents of children with autism (30). A back-translation procedure was employed to ensure linguistic and conceptual equivalence between the English and Chinese versions. In the current sample, the Affiliate Stigma Scale demonstrated excellent internal consistency, with a Cronbach’s alpha coefficient of 0.96 for the total scale.

#### 2.3.2 Health-related quality of life

HRQoL was assessed using the EQ-VAS and EQ-5D-3L questionnaire. The EQ-VAS captures the respondents’ self-rated health on a vertical visual analog scale ranging from 0 (worst imaginable health state) to 100 (best imaginable health state), reflecting their perceived health status at the time of assessment. Respondents indicate a numerical value on this scale corresponding to their current health perception. The EQ-5D-3L descriptive system comprises five dimensions: mobility, self-care, usual activities, pain/discomfort, and anxiety/depression. Each dimension has three severity levels: no problems, some problems, and extreme problems. In the current study sample, the Cronbach’s alpha coefficient of the EQ-5D-3L was 0.642. As this scale measures five distinct dimensions of health-related quality of life, each reflecting different aspects of health rather than a single latent trait, an alpha coefficient in the range of 0.6 to 0.7 is to be expected. In multidimensional health-related quality of life instruments, this level of internal consistency is generally considered acceptable. The self-reported responses across these dimensions were converted into a single health utility score using the Chinese EQ-5D-3L value set established in 2018 (31). This value was derived from population preferences using the time-trade-off method. The health utility score ranges from -0.1702 to 1.0000 and was calculated using the following formula:


Health Utility Score=1-(0.0766×MO2)-(0.2668×MO3)-(0.0441×SC2)-(0.2912×SC3)-(0.0370×UA2)-(0.0538×UA3)-(0.0274×PD2)-(0.0409×PD3)- (0.0539 × AD2)-(0.1771 × AD3) 

In this formula, MO2, SC2, UA2, PD2, and AD2 are dummy variables coded as 1 if the respondent reported level 2 problems (“some problems”) in mobility, self-care, usual activities, pain/discomfort, or anxiety/depression, respectively, and 0 otherwise. Similarly, MO3, SC3, UA3, PD3, and AD3 equal 1 if the respondent reported level 3 problems (“extreme problems”) in the corresponding dimensions, and 0 otherwise.

#### 2.3.3 Covariates

The following variables were included as covariates: age, sex, education, occupation, income, and work capacity. Age was categorized into three groups: ≥ 60 years, 45–59 years, and < 45 years. Education level was dichotomized as junior high school or below and above junior high school. Occupation was classified as full-time caregivers and non-full-time caregivers. Income was categorized into ≥ 34,325 RMB and < 34,325 RMB. In 2024, the per capita disposable income for all residents in Sichuan Province was 34,325 yuan (32). Work capacity was categorized into three levels: capable of work, partially capable of work, and incapacitated.

## 3 Statistical analysis

Statistical analyses were conducted using SPSS (version 27.0) and R software (version 4.3.3). Demographic characteristics of the participants are presented using frequencies and constituent ratios. Measurement data are presented as the mean ± standard deviation. Intergroup comparisons were conducted using the independent-samples t-test (for dichotomous variables, including gender and education level) or one-way analysis of variance (ANOVA) (for multicategorical variables, such as work capacity). If the one-way ANOVA indicated significant differences between groups, *post hoc* pairwise comparisons were performed utilizing the Games-Howell method. In this study, 142 participants (48%) had censored observations for EQ-5D-3L, and 18 participants (6%) had censored observations for EQ-VAS. Given the distributional characteristics of the HRQoL outcome variables, the Tobit regression model was applied to examine the association between affiliate stigma and HRQoL, as well as other influencing factors. The EQ-5D-3L utility index is censored at the upper bound of 1.0 (perfect health), with a certain proportion of respondents achieving this ceiling value. The Tobit model explicitly accounts for this censoring by estimating parameters using maximum likelihood, treating the observed data as a mixture of uncensored observations and observations censored at the upper limit. A p < 0.05 was considered statistically significant for all analyses.

## 4 Results

This study enrolled 297 primary caregivers of individuals with severe mental disorders. Among them, 159 were female (53.5%). In terms of age distribution, 55.9% were aged 60 years and above. Educational attainment was primarily junior high school level or below (85.2%). In total, 61.3% of caregivers provided full-time care. Annual income was mostly low, with 68.4% earning less than the per capita annual income of Sichuan residents. Over half of the sample (56.6%) remained within the working-age population. Detailed demographic information is presented in **Table 1**. The mean affiliate stigma score among caregivers was 48.67 (SD = 15.629). The mean HRQoL EQ-5D-3L utility index was 0.954 (SD = 0.067), and the mean VAS self-rated health score was 70.48 (SD = 17.22). Regarding the EQ-5D-3L dimensions, the highest proportion of caregivers reported problems in the “pain/discomfort” (37.7%), followed by anxiety/depression (36.7%), whereas the ‘self-care’ dimension had the lowest proportion reporting problems (3.0%) (**Supplementary Table A**).

**Table 1:** Caregiver demographic characteristics, affiliate stigma, and HRQoL.

Characteristics	n (%)		Health-related quality of life					
			EQ-5D-3L utility index			EQ-VAS Score		
		Mean ± SD	Mean ± SD	t/F	p	Mean ± SD	t/F	p
Gender				2.684	0.008**		1.277	0.203
Male	138 (46.5)		0.965 ± 0.057			71.86 ± 17.57		
Female	159 (53.5)		0.944 ± 0.079			69.30 ± 16.93		
Age		61.468 ± 13.294		3.332	0.037*		1.311	0.271
< 45	27 (9.1)		0.983 ± 0.025			75.11 ± 16.73		
45 - 60	104 (35.0)		0.958 ± 0.067			70.94 ± 18.94		
≥ 60	166 (55.9)		0.947 ± 0.076			69.45 ± 16.16		
Education				-1.463	0.144		-1.976	0.049*
Junior high and below	253 (85.2)		0.952 ± 0.072			69.66 ± 16.93		
Above junior high	44 (14.8)		0.968 ± 0.057			75.20 ± 18.48		
Income (RMB/year)		31355.604 ± 40950.943	-2.037	0.043*			-4.163	<0.001***
< 34,325	203 (68.4)		0.949 ± 0.074			67.72 ± 17.17		
≥ 34,325	94 (31.6)		0.965 ± 0.061			76.45 ± 15.95		
Full-time Caregiving Status				-0.763	0.446		-1.972	0.049*
Full-time	182 (61.3)		0.952 ± 0.074			68.92 ± 16.56		
Non-full-time	115 (38.7)		0.958 ± 0.064			72.96 ± 18.09		
Affiliate Stigma scores	297 (100)	48.670 ± 15.629						

Significance levels for P-values: *p < 0.05, **p < 0.01, ***p < 0.001.

Univariate analysis results (**Table 1** and relevant **supplementary materials**) revealed that gender, full-time caregiving status, age, education level, and income level were significantly associated with caregiver HRQoL (p < 0.05). Specifically, male caregivers exhibited significantly higher EQ-5D-3L utility index scores than female caregivers (p = 0.008). Non-full-time caregivers reported significantly higher VAS scores than full-time caregivers (p = 0.049). A significant difference was observed in the EQ-5D-3L utility index across age groups (p = 0.037). Games–Howell *post hoc* test results indicated that younger caregivers had significantly higher health utility scores than the middle-aged and older caregivers (**Supplementary Table B2**). Caregivers with higher education than junior high school reported higher VAS scores than those with junior high school education or less (p = 0.049). Caregivers with per capita annual income above disposable income in Sichuan Province demonstrated significantly higher EQ-5D-3L utility indices (p = 0.043) and VAS scores (p < 0.001) than those in the low-income group. No significant differences were observed in EQ-5D-3L utility indices or VAS scores across different working-age status groups (p > 0.05). Caregivers were further categorized into four quartiles (Q1 – Q4) based on total and subscale scores of affiliate stigma. ANOVA results (**Table 2** and relevant **supplementary materials**) indicated that irrespective of whether grouping was determined by total or subscale scores, affiliate stigma quartile groups exhibited significant differences in EQ-VAS scores and EQ-5D-3L utility indices (all p < 0.001). A decreasing trend in HRQoL indicators was observed with increasing levels of affiliate stigma quartiles.

**Table 2:** The status of health-related quality of life under varying levels of affiliate stigma.

ASS dimension	n(%)	EQ-VAS score (mean ± SD)	EQ-5D-3L utility index (mean ± SD)	ANOVA (EQ-VAS score)	ANOVA (EQ-5D-3L utility index)
Total ASS Score					
Q1 (≤ 38)	76 (25.6%)	76.6 ± 14.8	0.986 ± 0.027	F = 11.93***	F = 18.78***
Q2 (39 - 50)	76 (25.6%)	73.6 ± 16.4	0.972 ± 0.053		
Q3 (51 - 60)	74 (24.9%)	69.7 ± 17.2	0.944 ± 0.073		
Q4 (≥ 61)	71 (23.9%)	61.3 ± 17.1	0.912 ± 0.091		
Total (22 - 86)	297 (100%)	70.5 ± 17.2	0.954 ± 0.067		

ASS, Affiliate Stigma Scale. Significance levels for P-values: *p < 0.05, **p < 0.01, ***p < 0.001. Q1 – Q4 represent quartile groups based on the total ASS score.

To control for potential demographic and caregiving-related confounders, Tobit regression models were applied to examine the association between affiliate stigma and HRQoL (the results are presented in **Table 3**). After adjustment for covariates including gender, age, educational level, income, occupational status, and working-age status, affiliate stigma remained a significant negative predictor of the EQ-5D-3L utility index (*β* = - 0.0032, p < 0.001) and the EQ-VAS self-rated health score (*β* = - 0.3175, p < 0.001).For each one-point increase in the Affiliate Stigma Scale score, caregivers’ EQ-5D-3L utility index decreased by 0.0032 points. To provide a more intuitive understanding of this effect size, caregivers in the highest stigma quartile were compared with those in the lowest quartile, showing an estimated decrease in the utility index of 0.115 points. This reduction exceeds the minimal important difference of 0.05 for the EQ-5D-3L reported in previous studies. For the EQ-VAS, the regression coefficient was *β* = - 0.3175, indicating that a 36-point increase in stigma score was associated with an estimated 11.4-point decrease in self-rated health, a reduction that also exceeds the commonly cited minimal important difference of 7 points for VAS measurements. In the regression model predicting the EQ-5D-3L utility index, gender (female) was significantly associated with a lower utility index (*β* = - 0.0295, p = 0.028). Apart from gender, no other covariates were statistically significant in the EQ-5D-3L utility index model (p > 0.05). In the regression model for EQ-VAS scores, higher annual income (≥ 34,325 CNY) was a significant positive predictor of VAS scores (*β* = 7.6221, p < 0.001). Gender, age, education, occupational status, and working-age status exhibited no statistical significance (p > 0.05). The regression analysis results were largely consistent with the trends observed in the univariate analysis, with affiliate stigma remaining significantly associated after multivariable adjustment.

**Table 3:** Results of Tobit regression for EQ-5D-3L utility index and EQ-VAS score among caregivers of patients with severe mental disorders.

Variable	EQ-5D-3L utility index				EQ-VAS score			
	Coefficient	SE	P-value	95% CI	Coefficient	SE	P-value	95% CI
Affiliate Stigma	-0.0032	0.0005	< 0.001***	(-0.0041, -0.0023)	-0.3175	0.0641	< 0.001***	(-0.4431, -0.1919)
Gender (Ref: Male)	-0.0295	0.0134	0.0280*	(-0.0559, -0.0032)	-1.5755	1.9891	0.4280	(-5.4741, 2.3231)
Age Group (Ref: < 45 years)								
45–60 years	-0.0143	0.0268	0.5940	(-0.0669, 0.0383)	0.0932	3.7209	0.9800	(-7.1997, 7.3861)
≥ 60 years	-0.0223	0.0266	0.4020	(-0.0745, 0.0299)	-0.1078	3.7245	0.9770	(-7.4076, 7.1920)
Education Level (Ref: Junior high school and below)	0.0115	0.0205	0.5750	(-0.0286, 0.0516)	1.2162	2.9086	0.6760	(-4.4845, 6.9169)
Annual Income (Ref: < 34,325 CNY)	0.0186	0.0150	0.2140	(-0.0107, 0.0480)	7.6221	2.2112	< 0.001***	(3.2883, 11.9559)
Occupation (Ref: Not full-time)	-0.0046	0.0141	0.7430	(-0.0322, 0.0230)	2.1733	2.1004	0.3010	(-1.9434, 6.2900)
Work Capacity (Ref: Incapacitated)								
Capable of work	0.0240	0.0175	0.1700	(-0.0102, 0.0583)	2.9392	2.6545	0.2680	(-2.2635, 8.1420)
Partially capable of work	0.0011	0.0097	0.9110	(-0.0180, 0.0201)	1.2377	1.4948	0.4080	(-1.6922, 4.1675)

Significance levels for P-values: *p < 0.05, **p < 0.01, ***p < 0.001.

## 5 Discussion

Based on a multi-region sample from Sichuan Province, this study examined the association between affiliate stigma and HRQoL among primary caregivers of individuals with severe mental disorders. The findings indicate that the caregivers experienced moderate to high levels of affiliate stigma. Furthermore, after controlling for multiple demographic and caregiving-related factors, affiliate stigma remained significantly and negatively associated with HRQoL indicators. Additionally, gender (female) and lower annual household income were independently associated with poorer HRQoL indicators. Collectively, these findings suggest that affiliate stigma constitutes an essential social determinant influencing the health status of caregivers of individuals with severe mental disorders.

This study demonstrated a clear declining trend in caregiver HRQoL with increasing levels of affiliate stigma. This graded relationship was particularly evident in the ANOVA based on quartile groups, indicating that affiliate stigma does affect health at extreme levels and likely exerts a continuous effect across its entire distribution. This conclusion remained robust in the multivariable Tobit regression model. The finding is broadly consistent with previous findings that family members of individuals with mental disorders experience compromised physical and psychological health due to social stigma, role strain, and emotional burden, thereby reinforcing the central role of affiliate stigma in caregiver health research (14, 33). However, the mean EQ-5D-3L utility index observed among caregivers in this study was higher than levels reported in some comparable international studies (34). This discrepancy may arise from the relatively high proportion of older caregivers in our sample. With increasing age, caregivers may develop greater social acceptance of their caregiving role and enhanced self-efficacy, facilitating improved psychological adaptation. Additionally, cultural values in China that emphasize resilience and familial duty may contribute to underreporting of health problems (35, 36).

The association between affiliate stigma and HRQoL may be explained by multiple psychological and behavioral pathways. Affiliate stigma extends beyond negative cognitive appraisal and can elicit sustained emotional distress and behavioral withdrawal (37). On the psychological level, caregivers may internalize societal stereotypes surrounding mental illness during long-term care, leading to adverse emotional responses such as shame, self-blame, and helplessness. Prolonged exposure to these stressors has been linked to chronic stress responses in previous research, and may be associated with increased vulnerability to depression, anxiety, and somatic symptoms, which are in turn related to poorer subjective and objective health outcomes (38). However, Higher levels of stigma may be associated with avoidance and concealment strategies, which in turn are related to reduced social engagement and underutilization of formal and informal support resources. This social withdrawal may be associated with increased psychological burden and physical exhaustion, which are linked to diminished HRQoL (39). Moreover, affiliate stigma may be associated with adverse health-related behaviors among caregivers, such as delaying medical care or neglecting personal health needs, which are in turn linked to poorer long-term health status (40).

In the context of Chinese family collectivism, these mechanisms may be amplified. This study was conducted within the cultural context of Chinese family collectivism, where the individual and family are fundamentally interconnected. In this context, the stigma of mental illness spills over from the affected individual to the entire family unit. Collectivist norms may amplify this negative impact through four primary mechanisms (26, 38, 41, 42). First, caregivers who prioritize family harmony may be more likely to suppress emotions and avoid seeking help, which is associated with increased psychological burden. Second, caregivers concerned with preserving family reputation may be more inclined to conceal the illness, which is associated with loss of social support and isolation. Third, caregivers driven by filial responsibility may neglect their own health, which is associated with deterioration of physical and mental well-being. Fourth, in collectivist contexts, stigma may be experienced not merely as personal devaluation but as a collective threat to family identity, which is associated with more intense shame and guilt. Notably, collectivism is not monolithic, and urbanization is gradually transforming these traditional values. In some Western individualistic cultures, stigma threatens individual identity, whereas in collectivist cultures, it challenges the dignity of the entire family. Therefore, anti-stigma interventions must undergo cultural adaptation while encouraging individuals to confront the public, they must also strengthen intra-familial support and find solutions within a cultural framework that values harmony and face. Although we interpret the findings through the lens of family collectivism, this variable was not directly measured in the present study. Accordingly, the collectivism framework is used as an interpretive context rather than an empirically tested variable.

This study found that female caregivers had significantly lower EQ-5D-3L utility indices than their male counterparts, consistent with the findings of a previous study, which found that female caregivers are more susceptible to emotional burden and caregiving strain (43). Women often undertake more daily care and emotional labor within family caregiving, which is associated with a more sustained depletion of their psychological and physical resources and, consequently, lower health utility levels (44). Avargues-Navarro et al. reported that even within a female population, homemakers who also cared for individuals with Alzheimer’s disease experienced significantly higher levels of emotional exhaustion than those solely responsible for housework (45). Additionally, higher income was significantly associated with higher EQ-VAS self-rated health scores, indicating that economic resources may play a critical role in buffering caregiving stress and enhancing subjective health perceptions (46). An intersectionality analysis by Wemrell et al. using data from the Swedish National Public Health Survey further identified income as a key social determinant of self-rated health (47). Their study found that middle- and low-income groups had a significantly increased risk of poor self-rated health compared with high-income groups, regardless of gender and migration status. Adequate economic resources may be associated with better access to healthcare, reduced caregiving burdens, and strengthened coping capacity, which in turn are linked to more favorable perceptions of health (48).

In the multivariable analysis, associations between most demographic characteristics and HRQoL did not reach statistical significance after adjustment, except for gender and income. This pattern may be attributable to sample characteristics and model specification. Specifically, after controlling for affiliate stigma, the effects of some socio-demographic factors may be mediated or attenuated, indicating that affiliate stigma plays a more central explanatory role in caregiver health outcomes.

This study provides new insight into the health challenges faced by caregivers of individuals with severe mental disorders. However, the findings extend stigma research beyond patients themselves to include primary caregivers, thereby offering a more comprehensive understanding of the health burden associated with mental disorders at both familial and societal levels. Furthermore, this study demonstrated that in community mental health services and family-based interventions, focusing solely on symptom control for patients may be insufficient. Greater emphasis should be placed on caregivers’ psychological experiences, especially on identifying and mitigating affiliate stigma. Specifically, at the individual level, cognitive-behavioral interventions targeting stigma or acceptance and commitment therapy can be implemented to help caregivers identify and restructure internalized negative beliefs, reducing shame and experiential avoidance. At the family level, family psychoeducation incorporating stigma modules can be delivered to promote open communication and mutual support among family members. At the community level, anti-stigma public education can be conducted through trusted institutions such as community health centers, while training community workers to identify at-risk caregivers during home visits and provide initial support. At the policy level, it is recommended that psychological support for caregivers be integrated into routine community mental health services, and that support measures such as financial subsidies and respite care services be explored. Through the development of such a multi-level, culturally grounded intervention system, it is expected that caregiver stigma levels can be effectively reduced, thereby improving their health-related quality of life.

## 6 Study limitations

This study also has several limitations. First, the cross-sectional design precludes causal inference and does not allow us to determine the direction of the observed associations. While we hypothesized that affiliate stigma negatively affects HRQoL, it is also plausible that poorer HRQoL, characterized by greater psychological distress or physical health problems, may increase caregivers’ sensitivity to stigma or reduce their capacity to cope with stigmatizing experiences, thereby amplifying perceived affiliate stigma. This potential reverse causality cannot be ruled out with the current design. Longitudinal studies are needed to elucidate the temporal dynamics and potential bidirectional relationships between affiliate stigma and caregiver HRQoL. Second, the use of self-reported health measures may introduce measurement bias. Social desirability bias might have led caregivers to underreport affiliate stigma or overreport their health status, potentially resulting in conservative estimates of the association between stigma and HRQoL. Additionally, self-reported health measures, while widely used and validated, capture subjective perceptions that may be influenced by cultural norms, mood states at the time of assessment, and individual response styles. Future studies could benefit from incorporating objective health indicators or multi-informant reports to complement self-reported data. Third, although the multi-stage stratified sampling method employed in this study was designed to enhance representativeness by including diverse geographic regions and urban/rural areas within Sichuan Province, potential sampling biases still exist. The selection of six specific districts/counties, although stratified, may not fully capture the heterogeneity of all caregivers across the province, particularly those in remote mountainous regions or minority ethnic areas with distinct cultural practices. Future research should employ longitudinal or interventional designs to investigate the dynamic impact of changes in affiliate stigma on caregiver HRQoL. Incorporating more psychosocial variables, including coping strategies, social support, and family functioning, would help to develop more comprehensive theoretical models. Moreover, evaluating the effectiveness of interventions targeting affiliate stigma in improving caregiver HRQoL could provide more targeted evidence to inform community mental health policies and services.

## 7 Conclusions

This study demonstrated a significant negative association between affiliate stigma and HRQoL among primary caregivers of individuals with severe mental disorders in a collectivist cultural context. After controlling for key confounding factors including gender, age, and income, affiliate stigma remained a significant negative predictor of caregivers’ health-related quality of life, as assessed by both the health utility value (EQ-5D-3L) and self-rated health score (EQ-VAS).

These findings have important implications for mental health services and public health policy. First, the focus of mental health interventions should be extended from patients to family caregivers, with routine screening for affiliate stigma integrated into community services and targeted psychological support provided for caregivers at risk. Second, anti-stigma interventions need to fully consider cultural contexts, and public health campaigns should aim to reduce the stigmatization of mental illness while promoting greater social acceptance of families affected by mental disorders. Third, priority should be given to vulnerable groups, particularly female caregivers and those with limited economic resources, through policy measures such as financial assistance and respite care services to alleviate the dual burden of stigma and caregiving. Therefore, addressing affiliate stigma among caregivers should be recognized as a key priority in mental health services and public health policy.

## Data Availability

The raw data supporting the conclusions of this article will be made available by the authors, without undue reservation.
