# Supplementary material for: Affiliate stigma and health-related quality of life among caregivers of people with severe mental illness in a collectivist context: a cross-sectional study
Source: Front Psychiatry. 2026 Jun 12;17:1805823. doi: 10.3389/fpsyt.2026.1805823 (PMC13303785; doi:10.3389/fpsyt.2026.1805823)
Supplement: Supplementary file 1 [file Table1.docx]

Supplementary Material

**Table A.** Distribution of dimensions of affiliate stigma and health-related quality of life

| Tool & Dimension | Statistical Indicator / Level | Value |
| --- | --- | --- |
| **Affiliate stigma** |  |  |
| Cognitive Dimension | Mean ± SD | 15.39 ± 5.64 |
| Affective Dimension | Mean ± SD | 17.44 ± 5.68 |
| Behavioral Dimension | Mean ± SD | 15.83 ± 5.28 |
| **EQ-5D-3L** (n=297) |  |  |
| MOBILITY | No problems [n(%)] | 261 (87.88) |
|  | Some problems [n(%)] | 35 (11.78) |
|  | Extreme problems [n(%)] | 1 (0.34) |
| SELFCARE | No problems [n(%)] | 288 (97.00) |
|  | Some problems [n(%)] | 9 (3.00) |
|  | Extreme problems [n(%)] | 0 (0.00) |
| USUAL ACTIVITIES | No problems [n(%)] | 270 (90.91) |
|  | Some problems [n(%)] | 25 (8.42) |
|  | Extreme problems [n(%)] | 2 (0.67) |
| PAIN / DISCOMFORT | No problems [n(%)] | 185 (62.29) |
|  | Some problems [n(%)] | 110 (37.04) |
|  | Extreme problems [n(%)] | 2 (0.67) |
| ANXIETY / DEPRESSION | No problems [n(%)] | 188 (63.30) |
|  | Some problems [n(%)] | 93 (31.31) |
|  | Extreme problems [n(%)] | 16 (5.39) |

**Table B1.** Intergroup Comparisons and Games-Howell Post-Hoc Test Results Among Caregivers with Different Work Capacity

| Dependent Variable | Group Comparison | Mean Difference | Std. Error | P-value | 95% Confidence Interval | | |
| --- | --- | --- | --- | --- | --- | --- | --- |
|  |  |  |  |  | Lower Bound | | Upper Bound |
| **EQ-5D-3L Utility Index** | Capable vs Partially capable | 0.0148 | 0.0108 | 0.360 | -0.0109 | 0.0406 | |
|  | Capable vs Incapacitated | 0.0121 | 0.0106 | 0.485 | -0.0130 | 0.0373 | |
|  | Partially capable vs Incapacitated | -0.0027 | 0.0133 | 0.978 | -0.0343 | 0.0289 | |
| **EQ-VAS Score** | Capable vs Partially capable | 1.5546 | 2.5369 | 0.813 | -4.4625 | 7.5717 | |
|  | Capable vs Incapacitated | 3.1488 | 2.4226 | 0.398 | -2.6023 | 8.8999 | |
|  | Partially capable vs Incapacitated | 1.5942 | 2.9340 | 0.850 | -5.3638 | 8.5522 | |

Note: Work capacity is defined as: Capable of work ( n=168); Partially capable of work ( n=69); Incapacitated (n=60).

**Table B2.** Intergroup Comparisons and Games-Howell Post-Hoc Test Results Among Caregivers in Different Age Groups

| Dependent Variable | Group Comparison | Mean Difference | Std. Error | P  value | 95% Confidence Interval | |
| --- | --- | --- | --- | --- | --- | --- |
|  |  |  |  |  | Lower Bound | Upper Bound |
| **EQ-5D-3L Utility Index** | **Group 1 vs Group 2** | **0.025** | 0.008 | **0.009**** | **0.005** | **0.044** |
|  | **Group 1 vs Group 3** | **0.036** | 0.008 | **<0.001***** | **0.018** | **0.054** |
|  | Group 2 vs Group 3 | 0.011 | 0.009 | 0.416 | -0.010 | 0.032 |
| **EQ-VAS Score** | Group 1 vs Group 2 | 4.169 | 3.717 | 0.506 | -4.84 | 13.18 |
|  | Group 1 vs Group 3 | 5.665 | 3.456 | 0.243 | -2.80 | 14.13 |
|  | Group 2 vs Group 3 | 1.497 | 2.241 | 0.782 | -3.80 | 6.79 |

Note: Age groups are defined as follows: Group 1 (Young): ≤44 years; Group 2 (Middle-aged): 45-59 years; Group 3 (Older): ≥60 years. Significance codes: *p*<0 .05, **p < 0.01, ***p < 0.001.

**Complete Table 1.** **Caregiver Demographic Characteristics, Affiliate Stigma, and HRQoL**

| Characteristics | n (%) |  | | Health-Related Quality of Life | | | | | | | |
| --- | --- | --- | --- | --- | --- | --- | --- | --- | --- | --- | --- |
|  |  |  |  | EQ-5D-3L Utility Index | | | | EQ-VAS Score | | | |
|  |  | Mean ± SD | | Mean ± SD | | t/F | p | Mean ± SD | t/F | p |  |
| Gender |  |  | |  | | 2.684 | 0.008** |  | 1.277 | 0.203 |  |
| Male | 138 (46.5) |  | | 0.965 ± 0.057 | |  |  | 71.86 ± 17.57 |  |  |  |
| Female | 159 (53.5) |  | | 0.944 ± 0.079 | |  |  | 69.30 ± 16.93 |  |  |  |
| Age |  | 61.468 ± 13.294 | |  | | 3.332 | 0.037* |  | 1.311 | 0.271 |  |
| < 45 | 27 (9.1) |  | | 0.983 ± 0.025 | |  |  | 75.11 ± 16.73 |  |  |  |
| 45 - 60 | 104 (35.0) |  | | 0.958 ± 0.067 | |  |  | 70.94 ± 18.94 |  |  |  |
| ≥ 60 | 166 (55.9) |  | | 0.947 ± 0.076 | |  |  | 69.45 ± 16.16 |  |  |  |
| Education |  |  | |  | | -1.463 | 0.144 |  | -1.976 | 0.049* |  |
| Junior high and below | 253 (85.2) |  | | 0.952 ± 0.072 | |  |  | 69.66 ± 16.93 |  |  |  |
| Above junior high | 44 (14.8) |  | | 0.968 ± 0.057 | |  |  | 75.20 ± 18.48 |  |  |  |
| Income (RMB/year) |  | 31355.604 ± 40950.943 | | | | -2.037 | 0.043* |  | -4.163 | <0.001*** |  |
| < 34,325 | 203 (68.4) |  | | 0.949 ± 0.074 | |  |  | 67.72 ± 17.17 |  |  |  |
| ≥ 34,325 | 94 (31.6) |  | | 0.965 ± 0.061 | |  |  | 76.45 ± 15.95 |  |  |  |
| Full-time Caregiving Status |  | |  | |  | -0.763 | 0.446 |  | -1.972 | 0.049* |  |
| Full-time | 182 (61.3) |  | | 0.952 ± 0.074 | |  |  | 68.92 ± 16.56 |  |  |  |
| Non-full-time | 115 (38.7) |  | | 0.958 ± 0.064 | |  |  | 72.96 ± 18.09 |  |  |  |
| Work Capacity |  |  | |  | | 1.389 | 0.251 |  | 0.782 | 0.458 |  |
| Capable of work | 168(56.6) |  | | 0.960 ±0.065 | |  |  | 71.482 ±17.622 |  |  |  |
| Partially capable of work | 69(23.2) |  | | 0.945 ±0.080 | |  |  | 69.928 ±17.792 |  |  |  |
| Incapacitated | 60(20.2) |  | | 0.948 ±0.072 | |  |  | 68.333 ±15.532 |  |  |  |
| Affiliate Stigma scores | 297 (100) | 48.670 ± 15.629 | |  | |  |  |  |  |  |  |

Note: Significance levels for P-values: * p<0.05,** p<0.01，*** p<0.001.

**Detailed Table 2.The status of health-related quality of life under varying levels and dimensions of affiliate stigma**

| ASS Dimension | n(%) | EQ-VAS Score (Mean±SD) | EQ-5D-3L Utility Index (Mean±SD) | ANOVA  (EQ-VAS Score) | ANOVA  (EQ-5D-3L Utility Index) |  |
| --- | --- | --- | --- | --- | --- | --- |
| **Total ASS Score** |  |  |  |  |  |  |
| Q1 (≤38) | 76 (25.6%) | 76.6 ± 14.8 | 0.986 ± 0.027 | F=11.93*** | F=18.78*** |  |
| Q2 (39-50) | 76 (25.6%) | 73.6 ± 16.4 | 0.972 ± 0.053 |  |  |  |
| Q3 (51-60) | 74 (24.9%) | 69.7 ± 17.2 | 0.944 ± 0.073 |  |  |  |
| Q4 (≥61) | 71 (23.9%) | 61.3 ± 17.1 | 0.912 ± 0.091 |  |  |  |
| **Total (22-86)** | **297 (100%)** | **70.5 ± 17.2** | **0.954 ± 0.067** |  |  |  |
| **Cognitive Dimension** | | | | | |  |
| Q1 (≤11) | 79 (26.6%) | 76.2 ± 15.3 | 0.985 ± 0.028 | F=12.28*** | F=15.14*** |  |
| Q2 (12-15) | 71 (23.9%) | 76.0 ± 15.9 | 0.972 ± 0.055 |  |  |  |
| Q3 (16-20) | 77 (25.9%) | 66.0 ± 15.9 | 0.933 ± 0.078 |  |  |  |
| Q4 (≥21) | 70 (23.6%) | 63.4 ± 18.3 | 0.924 ± 0.087 |  |  |  |
| **Affective Dimension** | | | | | |  |
| Q1 (≤14) | 85 (28.6%) | 76.4 ± 14.6 | 0.986 ± 0.028 | F=11.06*** | F=28.18*** |  |
| Q2 (15-19) | 86 (29.0%) | 73.3 ± 16.2 | 0.972 ± 0.050 |  |  |  |
| Q3 (20-21) | 55 (18.5%) | 67.7 ± 17.0 | 0.947 ± 0.060 |  |  |  |
| Q4(≥22) | 71 (23.9%) | 62.2 ± 18.3 | 0.900 ± 0.097 |  |  |  |
| **Behavioral Dimension** | | | | | |  |
| Q1 (≤12) | 82 (27.6%) | 75.1 ± 15.5 | 0.980 ± 0.034 | F=10.45*** | F=9.60*** |  |
| Q2 (13-16) | 79 (26.6%) | 74.1 ± 17.6 | 0.964 ± 0.058 |  |  |  |
| Q3 (17-20) | 81 (27.3%) | 69.2 ± 17.2 | 0.934 ± 0.087 |  |  |  |
| Q4 (≥21) | 55 (18.5%) | 60.4 ± 15.1 | 0.929 ± 0.082 |  |  |  |

Notes: ASS = Affiliate Stigma Scale. Significance levels for P-values: * p<0.05, ** p<0.01, *** p<0.001. Q1–Q4 represent quartile groups based on the total ASS score and its subscale scores.
